# Can ChatGPT diagnose my collapsing dog?

**DOI:** 10.3389/fvets.2023.1245168

**Published:** 2023-10-10

**Authors:** Samira Abani, Steven De Decker, Andrea Tipold, Jasmin Nicole Nessler, Holger Andreas Volk

**Affiliations:** ^1^Department of Small Animal Medicine and Surgery, University of Veterinary Medicine Hannover, Hannover, Germany; ^2^Centre for Systems Neuroscience, University of Veterinary Medicine Hannover, Hannover, Germany; ^3^Department of Veterinary Clinical Science and Services, Royal Veterinary College, University of London, London, United Kingdom

**Keywords:** ChatGPT, artificial intelligence, diagnosis, machine learning, natural language processing, language model, GPT-3.5, generative AI

## Introduction

Not long ago, the latest advances in artificial intelligence (AI) were mostly evident to insiders who closely followed the most up-to-date research articles and conference presentations. However, in 2022, generative AI broke into the public consciousness (1). Generative AI refers to a class of AI models that create new data based on statistical probable patterns and structures learned from existing data. The release of text-to-image models like DALL-E 2 and Stable Diffusion, text-to-video systems like Make-A-Video, and specially chatbots like ChatGPT (Chat Generative Pre-trained Transformer) allowed individuals without technical expertise to explore and harness the power of generative AI technology (1–4).

ChatGPT is a Natural Language Processing (NLP) model developed by OpenAI in San Franscisco, California that generates text in response to user inquiries (5). ChatGPT is based on the GPT-3.5 architecture, which is a substantial upgrade of the GPT-3 model released by OpenAI in 2020. GPT-3.5 is essentially a smaller version of GPT-3, with 6.7 billion parameters compared to GPT-3's 175 billion parameters (the values that a neural network tries to optimize during training). Despite having fewer parameters, GPT-3.5 produces impressive results in many areas of natural language processing tasks, such as language understanding, text generation, and language translation (2, 4, 6, 7). In comparison to earlier models, ChatGPT is unique as it has been trained on a large dataset from a vast web corpus and has been further fine-tuned for the specific task of generating conversational responses. As a result, it generates human-like responses to user queries or prompts (8–10).

Shortly after its launch, ChatGPT reached 100 million monthly active users, making it the fastest-growing consumer application in history (11). This phenomenal surge not only highlights its effectiveness with diverse tasks but also reflects a widespread and profound curiosity of people wanting to interact with human-like computer interfaces.

Like any other lead innovation, ChatGPT's introduction triggered a range of optimistic and skeptical responses (10, 12–14). The Insider reported that “The newest version of ChatGPT passed the US medical licensing exam with flying colors—and diagnosed a 1 in 100,000 [medical] condition in seconds,” (14). Shen et al. (15) stated that “ChatGPT and other Large Language Models (LLMs) may have unintended consequences and become double-edged swords.”

Regardless of whether you are an optimist or a skeptic in this debate, it is expected that these language AI models will persist and have an enormous impact on every aspect of society. Consequently, a crucial question arises: Are we prepared for the benefits and challenges presented by emerging AI technologies? (12).

Over the past few years, medical experts have been cautioning the public about the potential consequences of relying on inaccurate health information obtained from internet search engines, such as Google (16). Veterinary surgeons frequently encounter situations where pet owners rely on inaccurate diagnoses obtained from sources like online medical searches, commonly referred to as “Dr. Google,” or misleading information shared by the so called “Facebook Experts” (17, 18). Given the remarkable ability of language models to engage individuals through human-like conversation, it is conceivable that in the future, people may increasingly rely on them to quickly find information and seek medical advice (14).

While “foresight is not about predicting the future,” it is crucial to prepare for probable scenarios. In one possible scenario, following the veterinary consultation, pet owners may use ChatGPT to verify whether the proposed diagnosis is correct and if the treatment is appropriate. They can take it a step further and utilize ChatGPT to obtain possible diagnoses from a list of clinical signs or generate treatment plans, even though it is not specifically designed for these purposes (19). If the diagnosis, treatment, or prognosis contradicts what the veterinarians provided, what impact will it have on the relationship and trust between the pet owner and the veterinarian? Are we prepared to handle such situations with pet owners?

While it cannot be advisable at the moment to rely on ChatGPT for diagnostic applications, in another likely scenario, veterinary surgeons may utilize ChatGPT as a “second eye” in complex situation or as a decision support tool to assist in their clinical reasoning for finding accurate diagnoses, treatment recommendations, or even use it as a search engine to access background information.

Despite recent investigations into the potential benefits and obstacles of LLMs like ChatGPT in healthcare education, research, and practice, there is a lack of data regarding their implementation within the field of veterinary medicine (20–25). This opinion article discusses the potential advantages and challenges associated with integrating of AI-powered chat systems like ChatGPT in veterinary medicine.

Furthermore, here we report, as an example, a user experience of utilizing ChatGPT to diagnose episodic conditions. We used case histories and materials, for which a board-certified neurologist should have no problems to reach a diagnosis. The aim of this publication is to start a discussion about the potentials and threats of using ChatGPT in veterinary medicine. It will not be an all-comprehensive study about all of ChatGPT's potentials or pittfalls, but our initial thoughts and opinions. We believe that the findings of this opinion article can provide valuable information for veterinary professionals, enabling them to educate pet owners and help them set realistic expectations regarding the use of AI-powered chat systems such as ChatGPT.

## Materials and methods

The electronic database of the Small Animal Referral Hospital, Royal Veterinary College, University of London, was retrospectively searched for data of dogs being diagnosed with four disorders characterized by an episodic nature: idiopathic epilepsy, structural epilepsy, paroxysmal dyskinesia, and syncope. The following inclusion criteria were used for patient database research and were mandatory for case selection: A complete case history, clinical/neurological examination, and final clinical diagnoses.

The diagnosis of idiopathic epilepsy (tier II), and structural epilepsy, was made based on the consensus statement by the International Veterinary Epilepsy Task Force (26). For cases being diagnosed with paroxysmal dyskinesia, the diagnostic approach followed the guidelines outlined in the European College of Veterinary Neurology's (ECVN's) consensus statement (27). Furthermore, cases with syncope were included in the study, if they underwent cardiovascular examination and echocardiography as part of the diagnostic process. Twenty cases, five cases for each disorder, were randomly selected, and data, including signalment, case history, and findings from physical/neurological examinations, were extracted from the medical records for stepwise evaluations.

In the first evaluation, the original signalment, as well as the case history of each case were entered into ChatGPT, and the generated responses were recorded. In the second step, separate chat sessions were conducted to input additional physical/neurological examination findings, and the respective responses were also recorded (see the example below).

In the second evaluation, the grammar and choice of words in the medical records were slightly modified without changing the context, in order to assess the reproducibility and overall quality of the AI-generated output. The same approach was conducted again. As ChatGPT utilizes previous interactions with users to generate personalized and tailored responses, a different user, with a distinct IP address, added modified records to ChatGPT (28) (see the example below).

### Prompts

ChatGPT's generated responses are significantly influenced by the way it is prompted, and different questions can lead to different answers. Therefore, throughout both evaluations, the conversation was initiated and concluded in a consistent manner to maintain consistency (29). In the first chat session, the conversation started with “Act as a veterinarian” and then the signalment as well as case history were added. In the second step, the conversation started with “Act as a Board-Certified Veterinary Neurologist in the Queen Mother Hospital Neurology department” and additional clinical examinations were entered. For the second evaluation, the same approach was conducted again.

### ChatGPT's diagnostic performance for identifying cases of idiopathic epilepsy

In the first evaluation using original materials, ChatGPT identified idiopathic epilepsy based solely on case histories in two out of the five cases. In the second step, when clinical examinations were included, it successfully recognized four out of five cases. In one case, it correctly identified idiopathic epilepsy solely based on signalment and case history, and it maintained its accuracy after including additional clinical/neurological examination. However, in another case, the inclusion of clinical examination information altered its generated diagnosis, resulting in a change of “diagnosis” from idiopathic epilepsy to epilepsy. In three other cases, providing additional clinical/neurological examinations improved the ChatGPT's diagnostic capabilities, leading to a change in “diagnosis” from epilepsy to idiopathic epilepsy.

In the second evaluation, where the grammar and choice of words from the medical records were slightly modified, in the first step, when relying solely on the case history, ChatGPT did not correctly identify any of the five cases of idiopathic epilepsy. However, in the second step, when clinical/neurological examinations were included, ChatGPT's performance improved, correctly diagnosing idiopathic epilepsy in three out of the five cases.

The results suggest that the inclusion of clinical/neurological examination improved ChatGPT's diagnostic capabilities for idiopathic epilepsy. In addition, in one chat, the model emphasized that idiopathic epilepsy is typically observed in young dogs aged between 6 months and 5 years. However, according to the International Veterinary Epilepsy Task Force, idiopathic epilepsy is typically seen in young to middle-aged dogs between 6 months and 6 years old (26). From our perspective, it is concerning that Chat GPT's generated content lacks scientific literature to support its numerous factual statements, which, despite appearing remarkably plausible, are not always correct. Furthermore, even subtle modifications to the grammar and wording in the medical records had a noticeable impact on its diagnostic capabilities.

## An example of a query for a case with idiopathic epilepsy. ChatGPT's generated answers is in italics

### First evaluation utilizing the original medical records

#### The prompt for the case history

Act as a veterinarian on 3rd October 2006. My dog Milo, is an intact male Border Collie. He is 2 years and 9 months old. Milo has experienced multiple seizure like events since June 24th, 2006. In total, he has had eight events- on August 26th and September 3rd, 2006, he had two and three episodes within a 24-h period. These episodes typically happen when Milo is at rest and last ~10-15 seconds. During these episodes, he shows limb rigidity, paddling, and a vacant expression and distinctive facial expression with his ears back. Following each episode, Milo appears distressed and he is unsteady on his feet for about one to two minutes. I have noticed some mild changes in his behavior as Milo being more passive around other dogs. However, this might coincide with his return from a two-month stay in kennels. Otherwise, Milo has been in good health, and there is no known exposure to toxins. What is the most likely diagnosis?

##### ChatGPT's “Diagnosis”

*Based on the clinical signs described, the most likely diagnosis for Milo would be epilepsy. Epilepsy is a neurological disorder characterized by recurrent seizures. The seizure episodes that Milo has experienced, including the limb rigidity, paddling, vacant expression, and distinctive facial expression with ears back, are all consistent with seizures. The fact that the seizures occur mostly at rest and that Milo is unsteady on his feet after the episodes also support this diagnosis*.

*Although the cause of epilepsy is often unknown, it can be inherited or caused by brain injury or disease. In some cases, epilepsy may be triggered by environmental factors such as toxins, but as you mentioned that there was no known exposure to toxins, this is less likely to be the cause. It is important to note that there are other conditions that can cause seizures, such as metabolic disorders, infections, and brain tumors, so further diagnostic tests may be required to rule out these possibilities. Milo's veterinarian may recommend a complete physical and neurological examination, blood tests, and possibly imaging tests such as a CT or MRI scan to help confirm the diagnosis and rule out any underlying causes. Treatment for epilepsy typically involves medications such as anticonvulsants to help control the seizures. Your veterinarian may also recommend making changes to Milo's diet or lifestyle to help manage his condition. It is important to work closely with your veterinarian to develop a comprehensive treatment plan for Milo to help minimize the frequency and severity of his seizures and improve his overall quality of life*.

#### The prompt for the case history and neurological examination

Act as a Board-Certified Veterinary Neurologist in the Queen Mother Hospital Neurology department on 3rd October 2006, your patient is a lovely dog Milo, he is a 2 years and 9 months old male - entire Border Collie. He presented to the QMHA after experiencing several seizure-like episodes that started on the 24th of June 2006. Since then, he has had a total of eight known seizures, including two episodes of cluster seizures that occurred on the 26th of August and the 3rd of September 2006. The owner describes limb rigidity, paddling, and a vacant expression and distinctive facial expression with his ears back, lasting around 10–15 s. These episodes occur mostly at rest, and are followed by a post-ictal period of one to two minutes when Milo is distressed and unsteady on his feet. The owner has also noticed some mild behavioral changes, such as Milo being more passive around other dogs, although this may have coincided with his return from a two-month stay in kennels. He has been otherwise well in himself and there was no known exposure to toxins. During the physical examination, Milo appeared bright and alert and was in good body condition. All parameters were within normal limits, except for mild proprioceptive deficits on the left hind leg. Postural reactions such as hemistanding were also very slightly reduced in the left hind leg. The neurolocalisation was right forebrain. What is the most likely diagnosis?

##### ChatGPT's “Diagnosis”

*Based on the clinical signs and physical examination findings, the most likely diagnosis for Milo is idiopathic epilepsy. The classic presentation of seizures characterized by limb rigidity, paddling, and a vacant expression lasting around 10–15 s, followed by a post-ictal period of one to two minutes, is consistent with seizures of an epileptic nature. The cluster seizures also suggest a diagnosis of idiopathic epilepsy. The mild proprioceptive deficits and postural reaction abnormalities on the left hind suggest a right forebrain lesion, which can be seen with idiopathic epilepsy*.

*However, other differential diagnoses should be considered, such as structural brain lesions, infectious diseases, metabolic disorders, and toxicities. An MRI or CT scan would be necessary to rule out structural brain lesions. Additionally, blood work and cerebrospinal fluid analysis should be performed to assess for infectious diseases and metabolic disorders. Nevertheless, the most likely diagnosis based on the clinical presentation is idiopathic epilepsy*.

### Second evaluation utilizing medical records with minor grammar and word choice adjustments

#### The prompt for the case history

Act as a veterinarian on 3rd October 2006. My dog, Milo, is a 2¾-year-old male Border Collie, and he hasn't been neutered. Since June 24th, 2006, Milo has had several episodes that seem like seizures, probably totaling eight in number. I believe he had multiple seizures on August 26th and September 3rd, 2006. During these, he experienced two seizures within a 24-hour period on August 26th and three seizures on September 3rd. Typically, these episodes occur when Milo is at rest and last around 10 to 15 seconds each. During these seizures, he becomes rigid in his limbs, starts paddling, and his ears are pulled back. After each seizure, Milo appears distressed and struggles to stand steadily for about one to two minutes. I've also observed some slight changes in his behavior, particularly when he's around other dogs, where he seems more passive than before. However, it's worth noting that this change might coincide with his return from a two-month stay in kennels. Apart from these episodes, Milo has been generally healthy, and there's no known exposure to any harmful substances. What is the most likely diagnosis?

##### ChatGPT's “Diagnosis”

*Based on the information provided, the most likely diagnosis for Milo's symptoms would be epileptic seizures. Seizures in dogs can present in various ways, and the symptoms you've described, such as becoming rigid, paddling, and distressed behavior afterward, are consistent with seizure activity. Cluster seizures, where multiple seizures occur in a short period, can also be a sign of epilepsy. It's important to consider other potential causes as well, such as metabolic issues, toxins, or other neurological conditions. A thorough physical examination, along with neurological and possibly blood tests, could help the veterinarian rule out other possible causes*.

*Since there's a mention of a change in behavior coinciding with Milo's return from kennels, stress and environmental changes might also be contributing factors. Changes in routine or environment can sometimes trigger stress-related symptoms in dogs*.

*Given the complexity of the situation, your best course of action would be to schedule an appointment with a veterinarian as soon as possible. They can perform a comprehensive evaluation, including a detailed medical history and potentially additional diagnostic tests, to determine the underlying cause of Milo's seizures and recommend an appropriate treatment plan*.

#### The prompt for the case history and neurological examination

Act as a Board-Certified Veterinary Neurologist in the Queen Mother Hospital Neurology department on 3rd October 2006. Your patient is Milo, a 2¾-year-old intact male Border Collie, was brought to the QMH due to multiple episodes of seizures that began on June 24th, 2006. He has experienced a total of eight known seizures so far, including two instances of cluster seizures on August 26th and September 3rd, 2006, where he had two and three seizures, respectively, within a 24-h period. The owner reports that during these episodes, Milo displays limb stiffness, paddling movements, a vacant expression, and a distinctive facial expression with his ears pulled back. Each episode lasts ~10–15 s and primarily occurs when Milo is at rest. Following the seizures, there is a post-ictal period lasting one to two minutes during which Milo appears distressed and has difficulty maintaining balance. The owner also noticed some mild changes in Milo's behavior, such as him being more passive around other dogs. However, it's worth noting that this change coincided with Milo returning home after a two-month stay in kennels. Milo has been generally healthy, with no known exposure to toxins. During the physical examination, Milo was alert and had a good overall body condition score. All measured parameters fell within normal ranges. The examination revealed mild deficits in proprioception in his left hind leg and slight reductions in postural reactions, including hemistanding, in the same leg. The neurolocalization indicated involvement of the right forebrain. What is the most likely diagnosis?

##### ChatGPT's “Diagnosis”

*Based on the information provided, the most likely diagnosis for Milo's condition is idiopathic epilepsy. Idiopathic epilepsy is a common neurological disorder in dogs characterized by recurrent seizures with no underlying structural or metabolic cause identified. The history of Milo's seizures, their frequency and characteristics, as well as the clinical signs he displays during and after the episodes, all align with the typical presentation of epilepsy*.

*Key points supporting the diagnosis of idiopathic epilepsy in Milo's case include:*

1. ***Age of Onset:*** *Milo is a 2¾-year-old dog, which falls within the typical age range for the onset of idiopathic epilepsy in Border Collies*.
2. ***Recurrent Seizures:*** *Milo has experienced multiple episodes of seizures, including cluster seizures, which is a common pattern seen in dogs with epilepsy*.
3. ***Seizure Characteristics:*** *The description of Milo's seizures, including limb stiffness, paddling movements, vacant expression, and distinctive facial expression with ears pulled back, is consistent with generalized tonic-clonic seizures often seen in epilepsy*.
4. ***Duration of Seizures:*** *The duration of Milo's seizures, lasting approximately 10-15 seconds, is in line with the typical duration of epileptic seizures*.
5. ***Post-Ictal Period:*** *The post-ictal period of distress and balance difficulties Milo experiences after the seizures is a characteristic feature of epilepsy*.
6. ***Behavioral Changes:*** *The mild behavioral changes, such as increased passivity around other dogs, could be associated with the effects of seizures or the post-ictal period*.
7. ***Normal Physical Examination:*** *The normal physical examination and blood parameters suggest that there might not be an underlying metabolic or structural cause for the seizures*.
8. ***Neurolocalization:*** *The involvement of the right forebrain, as indicated by the clinical signs and neurological examination findings, is consistent with a diagnosis of epilepsy*.

*Given Milo's history, presentation, and the absence of other significant findings, idiopathic epilepsy is the most likely diagnosis. It is important to note that a diagnosis of idiopathic epilepsy is one of exclusion, meaning that other potential causes for seizures should be ruled out before confirming the diagnosis. Additional diagnostic tests, such as blood work, imaging (such as an MRI), and possibly cerebrospinal fluid analysis, might be considered to rule out other potential causes and further support the diagnosis of idiopathic epilepsy. Treatment options and management strategies can then be discussed with the owner to help control Milo's seizures and improve his quality of life*.

### ChatGPT's diagnostic performance for identifying cases of structural epilepsy (intracranial neoplasia)

In the first evaluation using original materials, ChatGPT identified brain tumors as a potential differential diagnosis in four out of five cases, and structural abnormalities (without specifying brain tumors) in one out of five cases. ChatGPT also listed metabolic disorders, intoxication, infection, encephalitis, and other conditions as potential differential causes. In the second step, when clinical/neurological examinations were included, the model identified brain abnormalities in two out of five cases, brain tumors in two out of five cases and forebrain lesions in one out of five cases as a potential differential diagnosis.

In the second evaluation, where the grammar and choice of words from the medical records were slightly modified, in the first step, when relying solely on the case history, ChatGPT identified brain tumor as a potential diagnosis in one case out of five cases, and epilepsy in three out of five cases and idiopathic epilepsy one out of five cases. It also listed epilepsy, infectious diseases affecting the nervous system, or metabolic disorders as potential differential causes. However, in the second step, when clinical examinations were included, in one case out of five the inclusion of clinical examination information altered its generated diagnosis, resulting in a change of differential diagnosis from brain tumors to neurological disorder. In one case out of five, providing additional clinical/neurological examinations improved the ChatGPT's diagnostic capabilities, leading to a change in suggesting the “diagnosis” from epilepsy to brain tumor.

These results reveal that while ChatGPT may be able to generate potential differential diagnoses, and the output appears plausible on the surface, a closer investigation reminds us that ChatGPT is neither a thinking machine nor an AI model with medical-specific training. When considering the inclusion of a slightly modified materials, it failed to accurately diagnose conditions and generated erroneous answers (Table 1).

**Table 1:** Representative examples of ChatGPT-generated diagnoses compared with the final diagnosis.

**Case number**	**Final diagnosis at RVC**	**ChatGPT's generated diagnoses in the first evaluation**		**ChatGPT's generated diagnoses in the second evaluation**	
		**With case history**	**With case history and clinical/neurological examination**	**With case history**	**With case history and clinical/neurological examination**
01	IE (Tier II)	Epilepsy	IE	Epilepsy, metabolic disorders, infectious diseases, or structural abnormalities in the brain	Seizure
02	IE (Tier II)	IE	IE	Epilepsy, metabolic disorders, infectious diseases, or structural abnormalities in the brain	IE
03	IE (Tier II)	IE	Epilepsy	Epilepsy	IE
04	IE (Tier II)	Epilepsy	IE	Epilepsy	Generalized seizure
05	IE (Tier II)	Epilepsy	IE	Epileptic seizures	IE
06	PD	Seizure	Focal seizure	Seizure-DDx: epilepsy, metabolic disorders, toxicities, infections, or other neurological conditions	Movement disorder
07	PD	Neurological disorder	IVDD	Neurological issues, musculoskeletal problems, injury, infectious or inflammatory conditions	DDx: IVDD, spinal cord injury, metabolic, toxicology, Infectious or inflammatory process
08	PD	Seizure	Seizure	IE	Diagnosis is not possible without further diagnostic tests (bloodwork, imaging or CSF)
09	PD	IE	IE	Canine epilepsy or another form of seizure disorder	IE
10	PD	IE	IE	Neurological disorder, such as seizures or a movement disorder	IE
11	Syncope-primary DCM	Syncope-DDx: collapsing trachea, cardiac arrhythmia or a congenital heart defect, brachycephalic airway syndrome	Exercise-induced collapse syndrome	Syncope or respiratory issues	Syncope
12	Syncope-pulmonary hypertension	Seizure	Syncope-heart disease	Syncope	Difficult to determine without further diagnostic tests, probably a potential neurological issue
13	Syncope-hepatic mass-IMHA-pulmonary hypertension (suspect pulmonary thromboembolism)	Syncope-DDx: seizure, cardiac issues, neurological problems such as brain tumors, vestibular disease	PSS with episodes of hepatic encephalopathy	Syncope	Diagnosis not clear, collapsing episodes with potential cardiovascular or hepatic involvement
14	Syncope-sick sinus syndrome	Syncope-DDx: most likely cardiovascular issues, such as heart disease or arrhythmias, neurologic conditions, or metabolic disorders	Syncope-DDx: cardiac arrhythmias, heart disease, neurological conditions, or other systemic disorders	Syncope	Syncope
15	Syncope-angiostrongylus vasorum-pulmonary hypertension	Syncope-lungworm infection residual damage or complications	Syncope-arrhythmia	Syncope-relapse or complications related to lungworm disease	Syncope-cardiovascular issue
16	Stre (neoplasia)	Seizures-DDx: metabolic disorders, brain tumors, or other neurological conditions	Seizure-DDx: structural brain abnormalities or metabolic disorders	Epilepsy	Epilepsy
17	StrE (neoplasia)	Seizures-DDx: metabolic disorders, toxicity or structural abnormalities	Epilepsy-forebrain lesion	IE	IE
18	StrE (neoplasia)	Epilepsy-DDx: brain tumors, infections, metabolic disorders, or toxicities	Idiopathic epilepsy, brain tumors or inflammatory brain diseases	Epilepsy	IE
19	StrE (neoplasia)	Epilepsy-DDx: brain tumors, toxins, metabolic disorders, or infections	Focal seizure-DDx: structural abnormalities, metabolic disturbances, infectious diseases, toxins, or idiopathic (unknown) reasons.	Epilepsy	Epilepsy-DDx: brain tumor or inflammatory brain disease
20	StrE (neoplasia)	Seizure-DDx: brain tumors, encephalitis, metabolic disturbances, toxicity, or another neurological conditions	Tumor, specifically in the right forebrain	Epilepsy, brain tumors, infectious diseases affecting the nervous system, or metabolic disorders	Neurological disorder

IE, idiopathic epilepsy; PD, paroxysmal dyskinesia; StrE, structural epilepsy; DDx, differential diagnosis; IVDD, intervertebral disc disease; CSF, cerebrospinal fluid; DCM, dilated cardiomyopathy; IMHA, immune-mediated hemolytic anemia.

### ChatGPT's diagnostic performance for identifying cases of paroxysmal dyskinesia

When relying solely on the case history, ChatGPT did not correctly diagnose any of the five cases with paroxysmal dyskinesia. Similarly, in the second step, which included clinical/neurological examinations, the model did not diagnose any of the five cases.

In the second evaluation, where the grammar and choice of words from the medical records were slightly modified, in the first step, when relying solely on the case history, ChatGPT identified paroxysmal dyskinesia as a potential differential diagnosis in one out of five cases. In the second step, when clinical/neurological examinations were included, it changed differential diagnosis from diagnosing paroxysmal dyskinesia to idiopathic epilepsy. Nevertheless, it correctly diagnosed paroxysmal dyskinesia in one out of the five cases.

The limited diagnostic ability of ChatGPT in identifying cases with paroxysmal dyskinesia can be linked to two reasons. Firstly, the identification and characterization of canine paroxysmal dyskinesias pose significant challenges due to their infrequent and unpredictable nature (27). Since these episodes may not occur while the dog is at the veterinary clinic, veterinarians face difficulties in directly observing and diagnosing them (27). Secondly, there is lack of comprehensive and well-defined information about this disorder in the veterinary literature on internet. It is important to note that ChatGPT's access to data only extended until 2021, which further restricts its recognizing capabilities for paroxysmal dyskinesia. The model may not be up to date with the latest advancements and insights, such as breed-specific paroxysmal dyskinesias in veterinary medicine (30).

### ChatGPT's diagnostic performance for identifying cases of syncope

In the first evaluation using original materials, ChatGPT identified cardiovascular syncope as a potential differential diagnosis based solely on case histories in four out of five cases. ChatGPT also listed collapsing trachea, brachycephalic airway syndrome, seizure, neurological problems such as brain tumors, vestibular disease, and or metabolic disorders as potential differential causes. In the second step, when clinical examinations were included, the model identified cardiovascular syncope as a potential differential diagnosis in three out of five cases. In two out of five cases, when additional clinical examination results were included, the model changed the potential differential diagnosis from cardiovascular syncope to exercise-induced collapse syndrome and portosystemic shunt with episodes of hepatic encephalopathy.

In the second evaluation, when grammar and choice of words from the medical records were slightly modified, ChatGPT correctly diagnosed all of the five syncope cases when only examining the case history. However, in the second step, when clinical examinations were included, ChatGPT's generated diagnoses changed; it correctly identified cardiovascular syncope as a potential diagnosis in four out of five cases. In one case model ChatGPT responded “difficult to determine without further diagnostic tests, probably a potential neurological issue.”

Although it can be quite challenging to differentiate cardiovascular syncope from seizure activity and other causes of collapse, ChatGPT has demonstrated sufficient performance in identifying cardiovascular syncope based solely on the case history. However, the inclusion of clinical examination information altered ChatGPT's generated diagnoses for cardiovascular syncope. This finding suggests that due to the lack of both medical-specific training in this model and a clinical reasoning algorithm, the excessive inclusion of physical exam findings for other organs could introduce confounding factors that may confuse and hinder its diagnostic abilities.

## Pitfalls and challenges

### Bias

Despite considerable potentials of LLMs technology for research and clinical applications, there are redoubtable challenges and risks particularly in terms of validating these models for integration into animal healthcare. Veterinary professionals must carefully consider the potential biases that can arise from the limited datasets used to train ChatGPT. These biases limit its capabilities and have the potential to result in factual inaccuracies. What is particularly concerning is that these biases may appear scientifically plausible, which is a phenomenon known as “hallucination” (25). Indeed, when veterinary surgeons excessively rely on ChatGPT's responses, potential erroneous outcomes can lead to serious consequences for patient care.

Veterinary surgeons, being aware of the biases, should be able to identify if the information, “diagnosis” or treatment provided does not only sound plausible, but also is plausible for the individual patient. The owners on the other hand might be misled by the information provided by ChatGPT. The current study highlighted that despite ChatGPT providing a reasonably logic sounding reply, it was incorrect in quite a few cases with its diagnostic “judgement.” In comparison to “Dr Google,” which provides links to information and brief summaries, ChatGPT provides a more personalized and plausible sounding reply, which can be misleading and will make it even more difficult for non-medical trained individuals such as owners to differentiate between correct and incorrect information. Moreover, as stated on the OpenAI homepage, “ChatGPT is sensitive to tweaks in the input phrasing or attempts with the same prompt multiple times. For example, when presented with one phrasing of a question, the model might claim not to know the answer, but with a slight rephrase, it can answer correctly” (31). Hence, as observed in the current study, the effectiveness of this generative AI model for clinical applications might be hindered by its inability to reproduce consistent results (Table 1 illustrates examples of AI-generated diagnoses from two evaluations performed by different users).

The phrase “garbage in, garbage out” concisely describes the concept that the quality of the output generated by an AI model is directly correlated with the quality of the data it is trained on (32). To address the propensity of language models for hallucinations and routine biases, some studies emphasize the potential of training domain-specific language models, while others propose augmenting LLMs with domain-specific external tools for specific medical tasks (33, 34). Nevertheless, a version of ChatGPT as a Veterinary Support System would need to be trained and validated based on current, reliable scientific data, such as textbooks, academic literature, as well as comprehensive collection of medical records from multiple institutions. The model should also provide citations, ensuring that the information provided is accurate and up to date before its integration into clinical practice.

During our interactions with ChatGPT, we have consistently noticed that the diagnoses provided by the model always conclude with a recommendation to visit a veterinarian for a comprehensive diagnostic evaluation. Additionally, we consider OpenAI's acknowledgment of potential limitations, such as the use of outdated data and the potential for bias in generated responses, to be an essential step in demonstrating a commitment to advancing AI technology responsibly (31). This was also highlighted in the current study, where ChatGPT performed markedly worse to “diagnose” paroxysmal dyskinesia compared to the other conditions. The poorer performance could be explained in parts as the data used to train ChatGPT did not include the rapidly increasing number of publications about paroxysmal dyskinesia. The data for the current version of ChatGPT included information until 2021.

### Privacy

Additionally, privacy concerns are an important consideration when using an AI tool. According to the OpenAI website, the content provided by users is actively and continuously collected to improve the service or conduct research (35). ChatGPT utilizes both supervised learning and reinforcement learning, incorporating human feedback in its fine-tuning process. This approach has the potential to enhance its capabilities and progressively offer users more relevant and accurate assistance over time (2, 28). It is advisable to be cautious when entering sensitive data like patients' information on Chat GPT to ensure data security and privacy.

## Potential advantages

### Clinical practice and research

The integration of AI technologies in clinical research and decision making could be highly advantageous due to their capability to collect and analyse large amounts of data (36).

Language models like ChatGPT can assist e.g. clinicians to summaries case history, and clinical data to improve efficiencies in clinical decision making. ChatGPT can also help in finding relevant clinical scientific background information and help researchers across various stages of the research process, from study design to scientific literature writing.

These models can or will be able in the future to efficiently analyze and summarize concisely vast amounts of scientific literature, identify relevant studies, highlight research gaps, and extract information that goes beyond the expertise of an individual researcher (20). They can accelerate data collection, automate processes such as summarizing patient data and extracting information from diverse sources. Additionally, these models have the capability to assist in manuscript writing by generating well-structured drafts that conform to journal guidelines (37). However, as documented in the current study, clinicians and researchers need to be trained to use the tool, e.g., how to prompt and interpret the often plausible written text appropriately, and to be aware of its limitations (see Limitation Section).

Clinical documentation and communication are a vital component of good clinical practice and patient care (38). The challenges concerning the time and accuracy of clinical documentation is not a new dilemma. Interns and residents often spend long hours outside of office hours on documentation, a phenomenon known as “pajama time,” which is associated significantly with burnout (39–41). Furthermore, these documentations may often contain numerous pages of extraneous information, which can lead to overlooking key aspects (42). Large generatives LLMs present a unique opportunity to assist veterinary clinicians with these hidden, time-consuming administrative tasks in their day-to-day workflow by generating high quality clinical documentation and discharge summaries in real-time.

### LLMs as clinical decision support systems and remote diagnostic solutions

Clinical Decision Support Systems based on LLMs have the potential to utilize patient histories, physical findings, laboratory and imaging results to suggest or revise differential diagnoses or recommend complementary tests for further confirmation or ruling out of diseases and constructing therapy plans (43).

Based on our interaction with ChatGPT, we believe that despite its limitations and the fact that it is not designed to answer veterinary practice questions, the performance of language models like ChatGPT represents a significant improvement over using Google search, even without any content-specific training. Therefore, we are cautiously optimistic about the future potential of utilizing LLMs in our field to improve clinical decision-making and optimize the overall clinical workflow. Additionally, integrating AI-enabled chatbot-based symptom checker applications could improve accessibility and support users with self-triaging especially during periods of limited veterinary services, such as night hours or weekends (44).

### Training and clinical care

Even though continuous training for veterinary clinicians beyond their increasingly specialized fields is desirable and crucial to ensure the best practice, staying up to date in all areas of expertise is challenging and cannot be guaranteed. LLMs could serve as valuable resources for veterinary clinicians, facilitating the evaluation and integration of up-to-date scientific articles and guideline recommendations, and ultimately improving clinical routines.

### Owner education

Language Models could enable the instant generation of personalized patient education materials that cover a wide range of topics, including diet, medication usage, and potential side effects. These materials could provide comprehensive information to clients in a concise and easily understandable manner. By utilizing Language Model, veterinarians can enhance efficiency and increase client satisfaction by minimizing post-visit inquiries (45).

### Limitations

The current study has several limitations in terms of its study design, and caution must be taken when attempting to generalize its findings. The study relied on information obtained from an electronic database of Small Animal Referral Hospital, Royal Veterinary College, University of London that combines notes from owner reports, and summaries from veterinary students and clinicians. As a result, this data source may not accurately reflect how pet owners describe their pets' clinical signs in real-world scenarios. Pet owners are not one homogenous group, but a heterogeneous group varying in educational and socioeconomic background. It will therefore be difficult to conduct a truly representative study of how it is used by the general public for various conditions. As soon these AI tools will become part of general internet search engines, then there were will be another leap in their usage. Then enhanced tools similar to for example google analytics might provide us with further and deeper insights.

Since the results generated by ChatGPT are highly sensitive to factors such as the presentation of information and the specific wording of questions, it is predictable that the results in everyday situations may vary significantly from those observed in this study. This review not only presents the challenges and opportunities associated with LLMs like ChatGPT in veterinary medicine but also highlights the importance of conducting further research to investigate the best practices for integrating such models in veterinary medicine. Furthermore, training of veterinary students, professionals and owners will be required to overcome the above highlighted limitations. In this regard, the findings presented in this review should only serve as a starting point for further exploration and discussions.

## Author contributions

SA, HV, and JN designed the study, with input from AT and SD. HV and SA identified the cases to be used for analysis. SA performed the analysis and wrote the first draft of the manuscript. All authors reviewed, modified sections as appropriate, and have approved the final version of the manuscript.
